# Connectivity of stormwater ponds impacts Odonata abundance and species richness

**DOI:** 10.1007/s10980-024-01817-z

**Published:** 2024-02-28

**Authors:** I. C. Richmond, M. C. Perron, S. P. Boyle, F. R. Pick

**Affiliations:** 1 Department of Biology, University of Ottawa, 30 Marie Curie Private, Ottawa, ON K1N 6N5 Canada; 2 Department of Biology, Concordia University, 7141 Sherbrooke St. W., Montreal, QC H4B 1R6 Canada; 3 St. Lawrence River Institute of Environmental Sciences, 2 St. Lawrence Drive, Cornwall, ON K6H 4Z1 Canada; 4 School of Science and the Environment, Memorial University of Newfoundland - Grenfell, 20 University Dr, Corner Brook, NL A2H 5G5 Canada

**Keywords:** Anisoptera, Zygoptera, Urban ecology, Fragmentation, Circuitscape, Dispersal

## Abstract

**Context:**

The successful dispersal of an animal depends, partly, on landscape connectivity. Urbanization poses risks to dispersal activities by increasing hostile land cover types.

**Objectives:**

We investigated how connectivity of urban ponds impacted Odonata communities (dragonflies and damselflies), an order of semi-aquatic insects that actively disperse.

**Methods:**

We sampled 41 constructed stormwater ponds and 8 natural ponds in a metropolitan area. The effect of connectivity and the quantity of available adjacent habitats was tested at different scales for dragonflies (900 m) and damselflies (300 m), determined by a literature analysis, to account for differences in suborder dispersal capabilities.

**Results:**

Lower levels of connectivity and fewer nearest neighbours negatively impacted abundance, species richness, and composition of dragonflies (p values < 0.01, R^2^ = 0.18–0.70). Adult dragonfly abundance had a stronger positive relationship with connectivity than species richness. In particular, the abundance of adult dragonfly *Leucorrhinia frigida,* found almost exclusively at natural ponds, had a positive relationship with connectivity. Connectivity and the number of nearest neighbours had no significant impact on damselflies apart from a slight negative relationship between connectivity and species richness (p value = 0.02, R^2^ = 0.11). Natural ponds had significantly higher levels of connectivity when compared to stormwater ponds.

**Conclusions:**

Our results suggest that dragonflies are positively affected by increased connectivity in an urban landscape, with no benefit of connectivity to damselflies at the scale measured. We recommend intentional planning of urban stormwater pond networks, where individual ponds can act as stepping stones, incorporated with strategic inclusion of beneficial land cover types.

**Supplementary Information:**

The online version contains supplementary material available at 10.1007/s10980-024-01817-z.

## Introduction

Urbanization often alters or destroys natural habitats (Liu et al. 2016) and the subsequent changes have led to declines in wildlife populations and biodiversity worldwide (Elmqvist 2013). The high level of management and design by humans in cities results in urban landscapes which are spatially heterogeneous, interwoven with green and blue spaces of varying size and quality, such as private backyards and ponds in public parks (Gaston et al. 2013). When well designed and managed, green and blue spaces can provide habitat for a high diversity of wildlife and help mitigate the negative impacts of urbanization (Aronson et al. 2017). Although a city’s green and blue spaces are often not large enough to individually support large populations of wildlife, these pockets of habitat can act as “stepping stones” for individual plants and animals, improving the connectivity of the landscape and supporting diverse natural communities (Shanahan et al. 2011; Maynou et al. 2017).

Landscape connectivity is a crucial factor for many species’ survival and is a key element of landscape structure (Taylor et al. 1993; Fahrig and Merriam 1994). Landscape connectivity (hereafter connectivity) refers to the degree to which a landscape facilitates or impedes processes such as movement or dispersal of a species among resource patches (Taylor et al. 1993). A landscape with high connectivity and thus a high potential for movement among resource patches can facilitate higher species richness and abundance through the creation of a metapopulation (Ribeiro et al. 2011; Carrara et al. 2012). However, fragmentation of habitat does not always lead to decreased connectivity, as long as habitat patches are within a traversable distance and the amount of habitat is not decreased (Goodwin and Fahrig 2002; Fahrig 2003). Due to the high abundance of small, patchy habitats, urban areas have potential for creating ecologically important corridors and facilitating movement and dispersal between smaller populations within the city landscape (Fischer and Lindenmayer 2002; Gaston et al. 2013; Braaker et al. 2014; Iojă et al. 2014).

Urban blue spaces, such as rivers and ponds, are often quite rare when compared to green space. Blue spaces provide a myriad of services to urban inhabitants including, but not limited to, mental health benefits, cooling effects, and flood regulation (Hassall and Anderson 2015; de Bell et al. 2017; Hu and Li 2020). Stormwater ponds are urban blue spaces that are specifically designed to manage stormwater runoff and provide flood mitigation for city neighbourhoods. The difficulty and high cost of creating or restoring blue space makes the existing sources, such as stormwater ponds, highly valuable habitats within cities (Geist and Hawkins 2016). Despite their primary role in flood regulation, stormwater ponds provide habitat for urban plants and wildlife (Hassall and Anderson 2015; Perron and Pick 2020a), with comparable biodiversity of invertebrates and plants when compared to natural ponds within the same region in some cases (Hassall and Anderson 2015). Urban ponds are often distributed across a city and depending on their design and management can act as stepping stones for urban wildlife where small populations can persist, thus maintaining and promoting biodiversity (Hill et al. 2017, 2018; Oertli and Parris 2019).

Odonates are widely regarded as effective bioindicators for the quality of aquatic habitats (Corbet 1999, Smith et al. 2007; Villalobos-Jimenez et al. 2016), including in urban areas. Consisting of two suborders, Anisoptera (hereafter dragonflies) and Zygoptera (hereafter damselflies), Odonata have complex life histories that rely on the aquatic-terrestrial interface (Oertli 2008). Odonates are nymphs in their juvenile stage when they are aquatic and are significantly affected by both water quality and wetland plant community structure (Perron et al. 2021). In their final instar, odonate nymphs emerge into their adult terrestrial form. Adult dragonflies have strong dispersal abilities and disperse away from their natal habitats for reproduction (Corbet 1999), while adult damselflies are not as efficient at dispersal. Damselflies mostly remain at their natal habitats for reproduction, although some species have been reported dispersing to new habitats for reproduction (e.g., McPeek 1989).

Odonata dispersal activities can be impacted by a range of different landscape features. Many species will specialize in either lentic or lotic water bodies. Wetland species use lentic waters as their primary breeding grounds and will not reproduce in faster moving waters (Seidu et al. 2019). Wooded areas are used by odonates to rest, forage, and occasionally mate (Morton 1977; Fincke 1992). Grasslands and crops/pastures provide important cover for windy days and facilitate hunting (Corbet 1999). Odonates often perch on barren rock, sometimes using this land cover type to thermoregulate (McGeoch and Samways 1991). Sand and gravel land cover types can also act as important secondary habitat for odonates (Buczynski and Pakulnicka 2000). Both settlement and transportation are urban land cover types that impede odonate movement, resulting in higher rates of mortality when traversed (Samways and Steytler 1996; Soluk et al. 2011; Baxter-Gilbert et al. 2015; Córdoba-Aguilar and Rocha-Ortega 2019).

In recent years, the potential for individual manmade ponds in urban environments to act as habitat to a variety of taxa has been proven (Le Viol et al. 2012; Hassall 2014; Meland et al. 2020; Perron and Pick 2020a). Additionally, there is some evidence that stormwater ponds have the potential to facilitate connectivity among areas with disrupted ecosystems such as agricultural fields, highways, and cities (Minot et al. 2021; Clevenot et al. 2022; Šigutová et al. 2022; Birch et al. 2023; Liao et al. 2022). Connectivity matters for various aquatic taxa within cities, such as diving beetles (Liao et al. 2022), and invertebrates (Hyseni et al. 2021). However, it is still not clear how connectivity of urban stormwater ponds is influencing adult odonates, a key group for aquatic ecosystem health that are active dispersers (but see Minot et al. 2021 for the influence of pond connectivity on odonate nymphs). To address this gap, we tested how connectivity of the landscape surrounding urban ponds influences adult Odonata abundance, diversity, and species composition. Our objectives were to determine if, (1) the  connectivity surrounding ponds influences the odonate composition (2) the connectivity of stormwater ponds differs from that of natural ponds. We hypothesized that pond connectivity would impact odonate abundance, Shannon diversity, species richness, and species composition of Odonata at all ponds, due to the influence of connectivity on the ability of individuals to traverse the landscape and disperse. We predicted that all ponds with greater connectivity and more available habitat surrounding them would have higher abundance, species diversity, and species richness of dragonflies and damselflies due to increased ease of dispersal. We predicted that connectivity would influence species composition, with species that are stronger dispersers being more associated with higher levels of connectivity. Finally, we predicted that natural ponds with minimal surrounding development would have higher connectivity and thus higher abundance, Shannon diversity, and species richness than stormwater ponds.

## Methods

### Study sites

The study took place in the capital city of Canada (Ottawa, Ontario) with a population of 1.3 million covering an area of 2790 km^2^ (2015 data, City of Ottawa 2017). We selected 41 constructed stormwater ponds (out of total of ~ 150 ponds) based on two criteria; stormwater ponds were permanent water bodies and were ~1 ha in size. In addition, 8 natural ponds were selected within the city’s borders; these were no larger than 1 ha, naturally occurring and had < 1% impervious cover in their catchments. Lastly, all ponds were at least 1 km away from any rivers or lakes to minimize population overlap with riverine and lacustrine odonate species (Dolný et al. 2014).

We sampled 38 stormwater ponds and three natural ponds in the summer of 2015. In 2016, we sampled eight additional ponds (n = 3 stormwater ponds, n = 5 natural ponds) to increase sample size. We added stormwater ponds that increased the age range of our stormwater ponds and to ensure a more representative sample of the variation in landscape features across the city. The stormwater ponds ranged in age from 1 to 36 years post-construction. Stormwater ponds were in residential or commercial areas, whereas natural ponds were in more rural areas of the City of Ottawa (descriptive characteristics of each study pond are included in Supplementary Materials 1).

### Odonata sampling

We sampled adult dragonflies and damselflies twice at every study pond and all sampling was done by a single observer. We did not sample odonates on cloudy days, and we only sampled between 10:00 am and 4:00 pm, when temperatures were above 16 °C and wind speeds were below 30 km/h (as seen in Butler and DeMaynadier 2008). If wind conditions were above 10 km/h, we searched for sheltered individuals to ensure a complete survey as described in Butler and DeMaynadier (2008). We sampled twice in the season to ensure that we captured early and late flying species (Oertli et al. 2005). In 2015, we first sampled from June 13–July 26 and from July 27–August 28. In 2016, we sampled from June 16–July 5 and again from August 1–9. We conducted a 60 min survey at every study pond by walking around the perimeter of the pond, moving further from the waters’ edge for each rotation. Due to variation in the size of ponds, the number of rotations completed varied per site from one rotation to ten or more (as in Bried et al. 2007; Kadoya et al. 2008). We used the maximum abundance observed in a given rotation to avoid double-counting individuals between circuits at each pond. We identified individuals to species and if this was not possible from afar, we caught the individual in a sweep net and the species was identified using a hand lens and field guide (Jones et al. 2013). When we identified individuals by hand, we stopped the timer until the species was identified and released.

### Landscape resistance modelling

To quantify connectivity around focal ponds, we used resistance modelling via circuit theory in the program *Circuitscape* version 5.0 (Anantharaman et al. 2020). Circuit theory incorporates fragmented, small patches of good habitat within a landscape that has hostile land cover types, such as roads, as part of the measure of connectivity. Incorporating small scale patches into connectivity is critical in fine-scale highly heterogeneous environments such as cities. Circuit theory estimates the connectivity of a landscape by assuming different habitat types are more or less resistant to movement (McRae et al. 2008; Koen et al. 2014; Anantharaman et al. 2020). Unlike least-cost path models which have a single movement corridor, circuit theory allows connectivity modelling across landscapes by merging paths from many sites (i.e., nodes, Koen et al. 2014). In the program *Circuitscape* (McRae et al. 2008; Anantharaman et al. 2020), low resistance values are assigned to land cover types that best facilitate movement, i.e., “good” habitats, and high resistance values are assigned to land cover types that least facilitate movement, i.e., “hostile” habitats, to model connectivity across the landscape as electric current moving through a circuit (McRae et al. 2008; Koen et al. 2014). The model produces a landscape with mean current density values (analogous to mean animal movement density), which represents the functional connectivity for the study species. Modelling connectivity using graph theory, as is done in *Circuitscape*, has been identified as a potentially excellent way to inform management of pond networks (Hill et al. 2017).

The land cover data used in our resistance modelling was provided by the City of Ottawa (City of Ottawa 2011); these data were produced using aerial and LiDAR imagery collected in 2014 and were found to be accurate during our field sampling in 2015 and 2016. Manual updating of the land cover data was done for stormwater ponds that were constructed after 2014 and thus not initially shown in the land cover dataset. Land cover classes included crop/pasture, grassland, rock barren, sand/gravel, settlement, transportation, wetlands, water (urban water bodies such as lakes, golf course ponds, and stormwater ponds), large rivers (Rideau River and Ottawa River), and wooded area (10 classes in total, Table 1). We developed a landscape resistance scheme based on Odonata life history. Land cover was converted from a vector layer with a 1 m resolution to a raster with a resolution of 10 m. Simple low-medium–high resistance schemes have been shown to be effective across taxa (validated with amphibians, reptiles, and fishers in Koen et al. 2014). Following Koen et al.’s (2014) methodology, all land cover classes were assigned low (1), medium (10), or high (100) resistance (Table 1). Low resistance land cover were classes that are primary habitat, medium resistance were classes that provide resources but are not main habitat, and high resistance land cover were classes that are difficult to traverse and cause mortality. Using evidence from the literature, wetlands and water were classified as low resistance, since they are the primary habitat for Odonata reproduction in this study (Table 1, Corbet 1999). Crop/pasture, grassland, large rivers, barren rock, sand/gravel, and wooded area were all classified as medium resistance as odonates can use them for resources (Table 1). The urban land use types settlement and transportation were classified as high resistance (Table 1, Boyle et al. 2017).

**Table 1:** Land use types and their resistance classification based on odonate biology for use in Circuitscape

Land use type	Resistance classification	Value in Circuitscape	References
Crop/pasture	Medium	10	Corbet (1999), Hernandez et al. (2006)
Grassland	Medium	10	Corbet (1999)
Large rivers	Medium	10	Dolný et al. (2014)
Rock barren	Medium	10	McGeoch and Samways (1991)
Sand/gravel	Medium	10	Buczynski and Pakulnicka (2000)
Settlement	High	100	Samways and Steytler (1996), Córdoba-Aguilar and Rocha-Ortega (2019)
Transportation	High	100	Soluk et al. (2011), Baxter-Gilbert et al. (2015)
Wooded area	Medium	10	Morton (1977), Fincke (1992)
Wetlands	Low	1	Hassall and Anderson (2015), Perron and Pick (2020a)
Water	Low	1	Dolný et al. (2014)

Classifications were made based on the scientific literature cited here

**Fig. 1:**
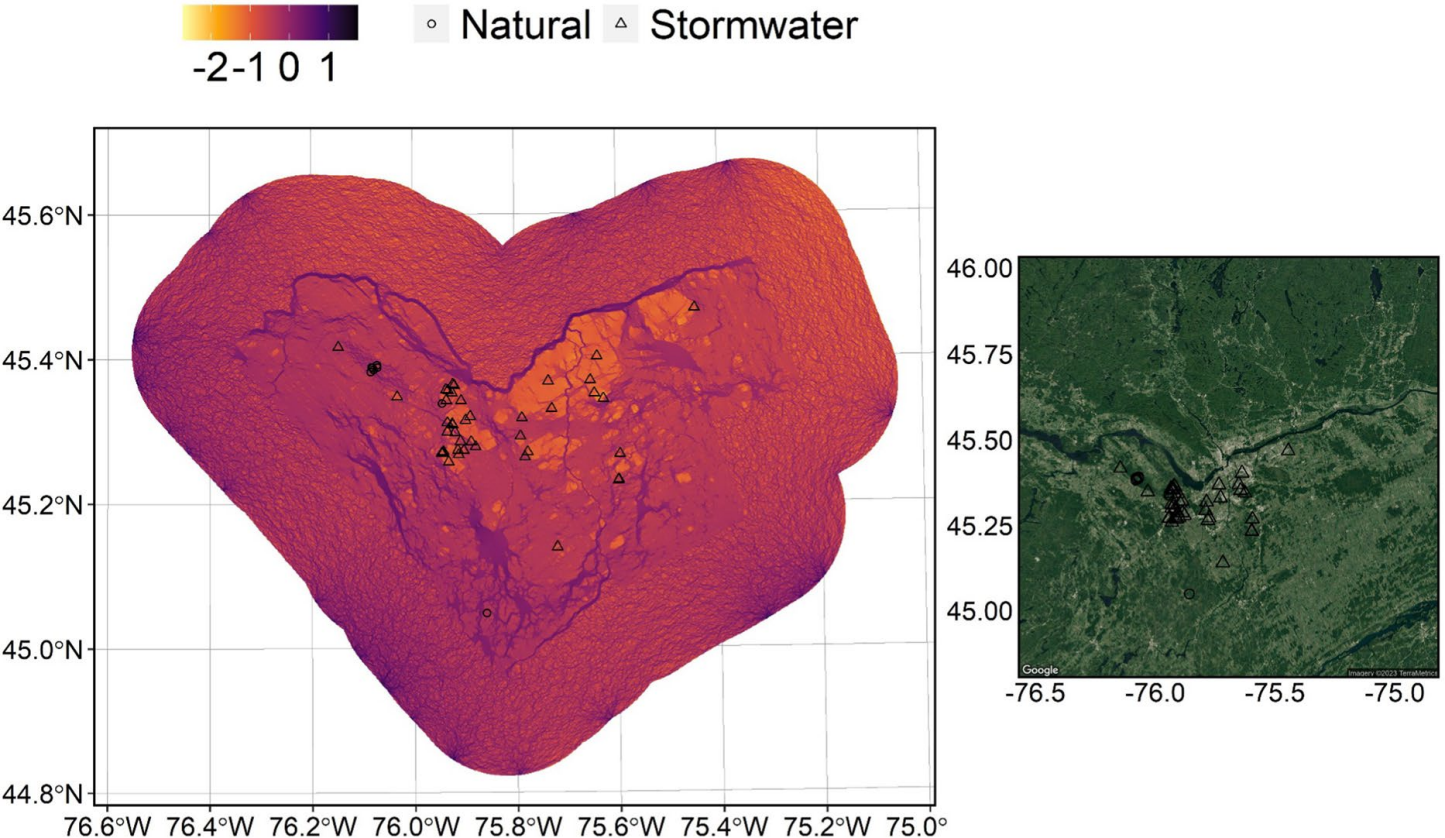
Left: Mean current density map of Ottawa, Ontario, surrounded by buffer with a randomized distribution of resistances and resolution of 10 m. Landcover data were based on a combination of aerial photography and LiDAR imagery (City of Ottawa 2011). Current (i.e., connectivity) was calculated using Circuitscape V 5.0, with 50 nodes in the pairwise, 8 neighbour mode. Higher current values indicate greater connectivity and imply potential for higher use of such habitats by pond odonates. Right: Study area map with satellite imagery. Satellite imagery retrieved from Google Maps on September 13, 2023. Location geocode used was “Ottawa, Ontario”. Both: Geographic coordinate system is WGS84 (epsg: 4326). Locations of natural ponds (n = 8) are denoted by circles (•) and the locations of stormwater ponds (n = 41) are denoted by triangles (▴). Shapes are hollow and overlapping to facilitate seeing sites that are close together, but no ponds are truly overlapping in situ. Figure created in R version 4.1.0 (R Core Team 2020)

Maps with artificial boundaries can overestimate resistance values in a landscape because the edges on a map do not represent the edges of habitat on the ground (Koen et al. 2010). To account for this and improve accuracy, we produced a 15 km buffer surrounding our study area, i.e., > 20% of the total study area, that had a random but representative ratio of resistance values (i.e., same ratio of low-medium–high as study area, 14.2%:34.8%:51.0%) and randomly generated 50 nodes around the perimeter (Koen et al. 2010). We then ran *Circuitscape* in pairwise-mode, using 8 neighbours. In total, 1225 pairs of nodes were created, with current pathways between each node and its nearest 8 neighbours was calculated and averaged to create a mean current map (Fig. 1).

### Scales of analysis

We performed a systematic literature review to determine the appropriate scales of analysis for dragonflies and damselflies, respectively. Following the PRISMA framework (Page et al. 2021), we used 9 keyword combinations in Scopus and reviewed a total of 293 scientific articles. We scanned the abstracts of all returned results to determine if they were relevant to our study. Specifically, we were searching for studies that reported tracked daily odonate movement distances measured and removed any papers that did not report tracked distances, and/or tracked migratory flights, only measured to a certain distance, duplicated data from another study, simulated flight data, or had flight measurements that were measured over a longer time period. After filtering the abstracts, we obtained 50 individual papers that appeared to have unique data and reported daily tracked flight distances of odonates. Out of the 50 papers, 1 could not be accessed using Scopus or Google Scholar. The 49 remaining papers were then read in-depth to extract study species and mean and maximum flight distances, when available. A total of 22 of the 50 papers had usable data and we extracted all mean and maximum distances (averaged across study years and/or sites when presented separately). The keywords used, results at each stage, and a summary table of the distances we found for dragonflies and damselflies can be found in Supplementary Material 2. We selected the maximum mean value rounded to the nearest 100 m for our subsequent analyses, resulting in a scale of 900 m for dragonflies and 300 m for damselflies.

### Statistical analyses

We used estimated abundance, Shannon diversity, and species richness as response variables. We calculated estimated site abundance by summing the total number of observations of each species at a given pond. We calculated species richness by counting and summing the number of species observed at a given pond. Lastly, we calculated the Shannon diversity using the “diversity” function from the package *vegan,* at a given pond (Oksanen et al. 2020). All statistical analysis was executed in R version 4.1.0 (R Core Team 2020).

We tested three explanatory variables in our analysis: mean current density, standard deviation of current, and number of nearest neighbours. Mean current density is a proxy for connectivity, with higher levels of mean current density indicating easier movement and more connectivity. To obtain measurements of mean current density, we used the “extract” function in the *raster* package to extract all current values in 900 m and 300 m zones around each of our study ponds (Hijmans et al. 2020). After extracting all values within the specified 900 m and 300 m areas, we calculated the mean for each study pond at both scales. We then calculated the standard deviation of the mean current for each study pond at both scales. To calculate number of nearest neighbours, we retrieved coordinates of all wetlands, small ponds, and stormwater ponds throughout the city. We did so by calculating the centroid of all “wetland” and “water” polygons (excluding rivers) in the land cover layer, using the “st_centroid” function in the *sf* package (Pebesma et al. 2021). Rivers were excluded because the odonate species we are studying are pond dwellers and do not use rivers as their breeding habitats (Dolný et al. 2014). All stormwater management facility locations in the city at the time of sampling were provided by the City of Ottawa (n = 492 total, City of Ottawa 2015). We extracted all active stormwater pond coordinates from the dataset provided and used them in our analysis (n = 188/492). We then produced 900 m and 300 m buffers around each of our study ponds using the “st_buffer” function in the *sf* package and tallied the total number of nearest neighbours within each buffer for all our study ponds.

We used general linear models to test the relationships between our variables. After running general linear models with all three explanatory variables, we determined that standard deviation of current was acting as an uninformative parameter (i.e., it is a variable with no relationship to the response variable and its addition to the models did not improve the log-likelihood of the model) and was thus removed from all analysis (Leroux 2019). Mean current and number of nearest neighbours were highly correlated (R = 0.75 at 900 m, R = 0.58 at 300 m), so we ran two separate models for every response variable. Estimated abundance, Shannon diversity, and species richness were each modelled with mean current and number of nearest neighbours separately at the 900 m and 300 m scales, resulting in a total of 12 models (six each for dragonflies and damselflies). The “lm” function with a Gaussian error structure was used to execute our models. Residuals were checked for assumptions of normality, independence, and homogeneity before analyzing our results.

To test the relationship between mean current and nearest neighbours with species composition, we performed transformation-based redundancy analyses (tb-RDA) with a Hellinger transformation, using the *vegan* package (Oksanen et al. 2020). Hellinger transformations downweight the zeroes in the dataset (Legendre and Gallagher 2001). We tested mean current density and number of nearest neighbours for relationships with dragonfly species composition and damselfly species composition. We used permutation tests (n = 999) and regressed the relationships to test statistical significance between mean current density and number of nearest neighbours with community composition of dragonflies and damselflies. We calculated goodness of fit (adjusted R^2^) for each model and conducted permutation tests for constrained analysis to determine the significance of the models and the redundancy axes with the *vegan* package. We plotted data using scaling type 2, which is appropriate when analyzing the differences among communities.

## Results

### Current map

The current density map generated by *Circuitscape* (Fig. 1) is an estimate for animal movement throughout the study area, where areas of higher mean current density correspond to high likelihood of animals moving through them resulting from the spatial patterning of high-quality habitat (i.e., areas with low resistance). Thus, in this context, our results can be interpreted by considering high current density an analog for high connectivity. Our current map (Fig. 1) displayed no effect of current buildup within the study area, indicating that the randomized buffer surrounding our study area successfully negated the effect of overestimation of current resistance caused by artificial edges created by map boundaries. Thus, our map meets the threshold for analysis as outlined in Koen et al. (2014). As expected, corridors of current were apparent through natural areas, especially wetland complexes. The effect of wetland complexes on connectivity, i.e., mean current density, can be seen in the northwest area of the study area, where the natural study sites are found (Fig. 1). The natural study sites are found in an area with a high density of wetlands and there are corresponding high values of mean current density (Fig. 1). The effect of wetlands on connectivity was emphasized through natural areas bordered by large swaths of developed land, such as the land surrounding stormwater ponds where the width of the high current area shrunk to pinch points. Conversely, when examining the highly developed areas of the landscape with a lot of settlement and transportation, such as in the northeast, there are lower values of mean current density, indicating lower connectivity (Fig. 1). The areas with the highest current were in the southern portion of the study area, located outside of the main urban centre, and were comprised of a mix of farmland and low disturbance wetland/upland spaces (Supplementary Material 1).

### Effects of current and surrounding habitat on Odonata

**Fig. 2:**
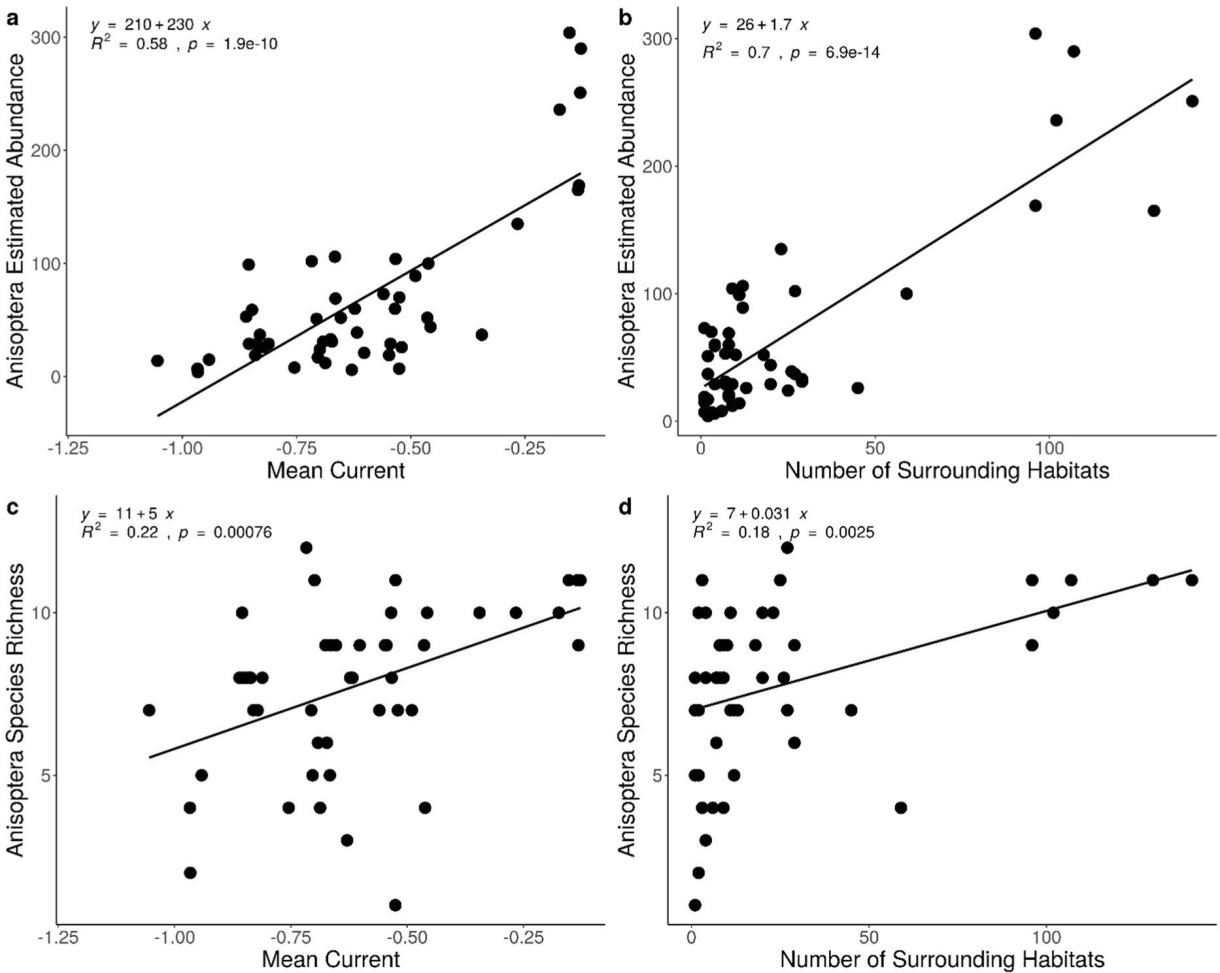
**a** Relationship between dragonfly (Anisoptera) estimated abundance and mean current density. **b** Relationship between dragonfly estimated abundance and number of surrounding habitats. **c** Relationship between dragonfly species richness and mean current density. **d** Relationship between dragonfly species richness and number of surrounding habitats. Lines and equations represent linear regression. All relationships were significant (p < 0.001). Mean current was calculated by averaging the pixel values within 900 m of every study site and number of nearest neighbours was calculated by tallying the number of other wetlands and stormwater ponds found within 900 m of every study site. Figure created in R version 4.1.0 (R Core Team 2020)

**Fig. 3:**
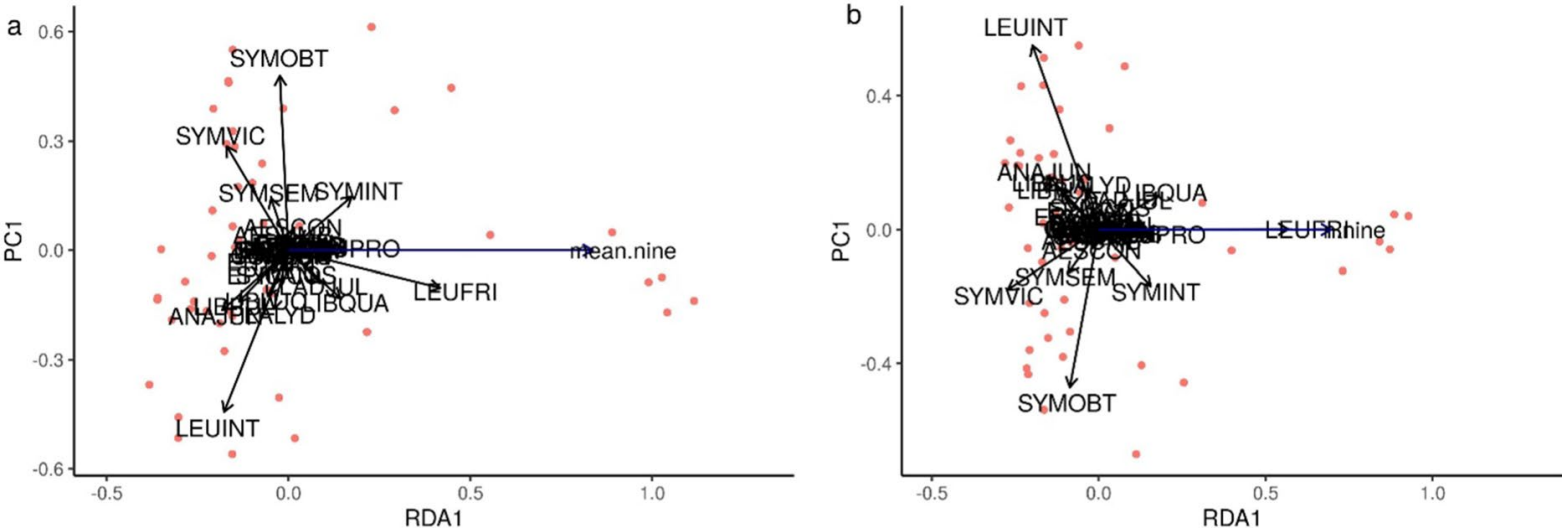
Transformation based-redundancy analysis (tb-rda), using a Hellinger transformation, of the relationship between adult dragonfly species composition (black vectors without arrows) **a** mean current at a 900 m scale (mean.nine), **b** number of nearest neighbours at a 900 m scale (n.nine) at urban ponds (n = 49). Figure created in R version 4.1.0 (R Core Team 2020)

Dragonfly estimated adult abundance and species richness were significantly and positively related to mean current density and number of habitats surrounding the study sites. Mean current density had a highly significant relationship with estimated abundance of dragonflies (R^2^ = 0.58, *p* = 1.92 × 10^–10^, Fig. 2a). The number of nearest neighbours explained more of the variation (R^2^ = 0.70*, p* = 6.94 × 10^–14^, Fig. 2b). Mean current density and number of nearest neighbours had significant relationships with dragonfly species richness (R^2^ = 0.22 *p* = 7.59 × 10^–4^, Fig. 2c; R^2^ = 0.18,* p* = 2.47 × 10^–3^, Fig. 2d). However, Shannon diversity was not significantly related to either of the variables tested. There was a weak but significant relationship between dragonfly species composition and mean current density (R^2^_adj_ = 0.077, F = 4.99, p = 0.002) as well as number of nearest neighbours (R^2^_adj_ = 0.099, F = 6.28, p = 0.002). Only one specific species of dragonfly was strongly associated with mean current density and number of nearest neighbours, *Leucorrhinia frigida* (Fig. 3). The dragonfly *Leucorrhinia frigida* had a positive linear relationship with mean current and number of nearest neighbours (Fig. 3). Model summary tables can be found in Supplementary Material 3.

**Fig. 4:**
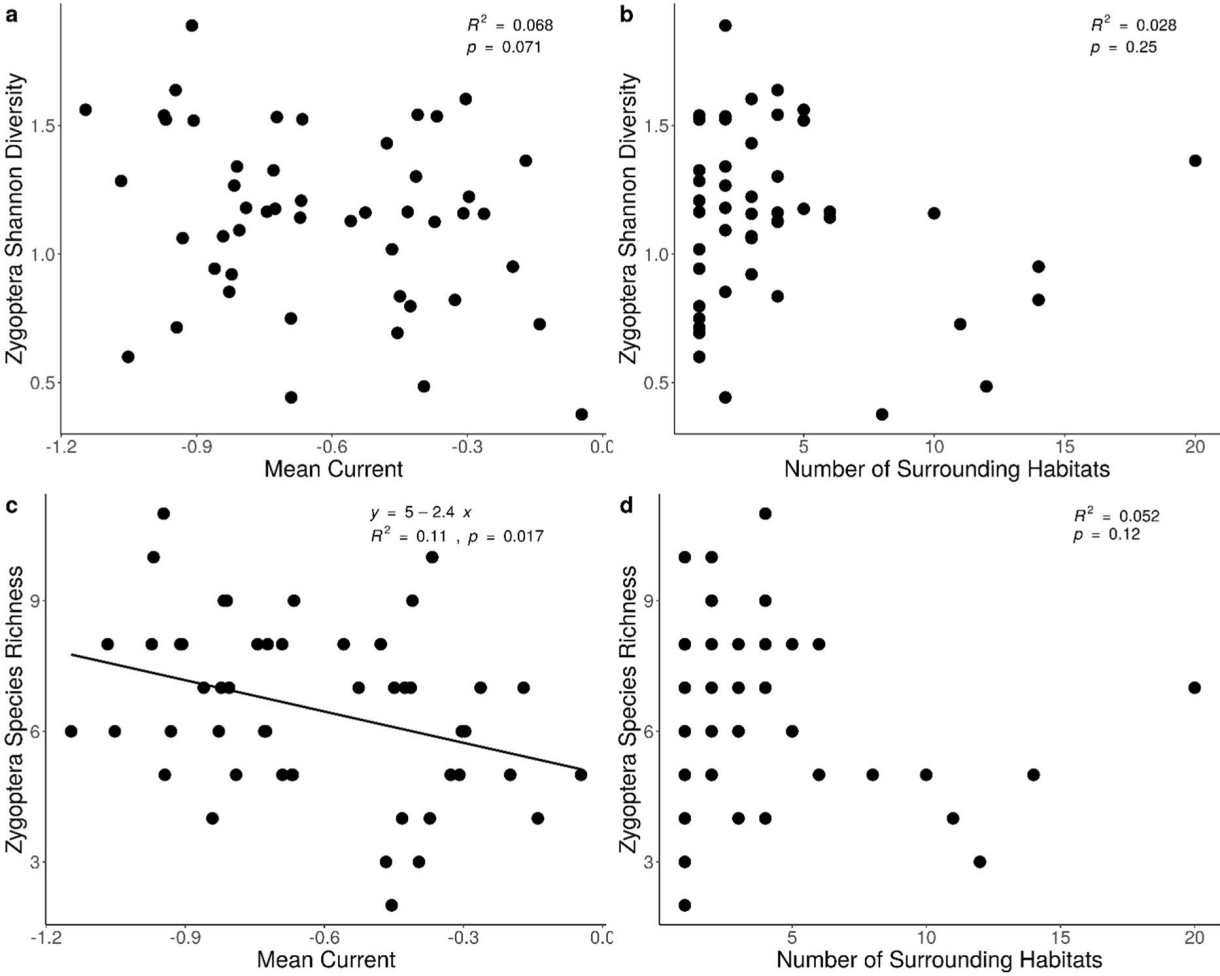
**a** Relationship between damselfly (Zygoptera) Shannon diversity and mean current density. **b** Relationship between damselfy Shannon diversity and number of surrounding habitats. **c** Relationship between damselfly species richness and mean current density. **d** Relationship between damselfly species richness and number of surrounding habitats. Lines and equations represent linear regression for statistically significant relationships. Mean current was calculated by averaging the pixel values within 300 m of every study site and number of nearest neighbours was calculated by tallying the number of other wetlands and stormwater ponds found within 300 m of every study site. Figure created in R version 4.1.0 (R Core Team 2020)

Damselfly estimated abundance did not have any significant relationships at the scale measured. Damselfly Shannon diversity and species richness were both negatively related to mean current density. However, the relationship between damselfly Shannon diversity and mean current density was marginal (R^2^ = 0.06, *p* = 0.071, Fig. 4a). The relationship between damselfly species richness and mean current density was significant (R^2^ = 0.11, *p* = 0.017, Fig. 4c). Number of nearest neighbours did not have any significant relationships with damselfly estimated abundance, Shannon diversity, or species richness. There was a significant relationship between damselfly species composition and mean current (R^2^_adj_ = 0.063, F = 4.24, *p* = 0.004) as well as number of nearest neighbours (R^2^_adj_ = 0.11, F = 6.65, *p* = 0.002), but explanatory power was lower than what was seen for dragonflies. There were no clear relationships between specific damselfly species and the mean current (Fig. S4-1) or the number of nearest neighbours (Fig. S4-2, Supplementary Material 3 contains full model details and Supplementary Materia 4 contains those for damselfly redundancy analyses).

### Natural vs. constructed stormwater ponds

**Fig. 5:**
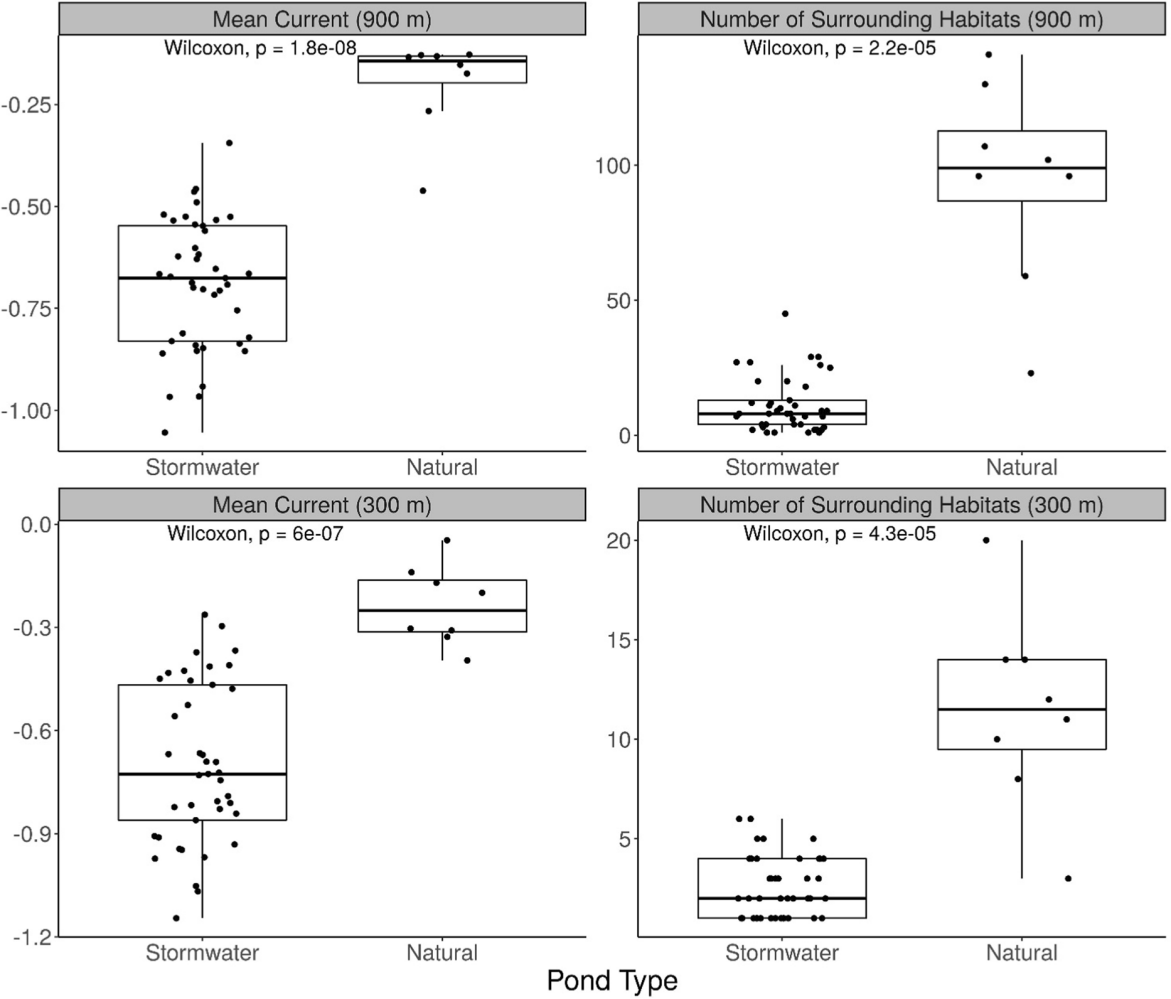
Comparisons between stormwater (n = 41) and natural ponds (n = 8) using a Wilcoxon rank-sum test for all statistically significant explanatory variables. The 900 m scale was used for dragonfly analysis and the 300 m scale was used for the damselfly analysis. Figure created in R version 4.1.0 (R Core Team 2020)

At the 900 m scale, mean current density values at stormwater ponds ranged from − 1.05 to − 0.34 (mean = − 0.69 ± 0.025 SE) and the number of nearest neighbours, calculated by summing wetlands, stormwater ponds, and small ponds within the 900 m buffer zone, ranged from 1 to 45 (mean = 11 ± 1.58, Fig. 5). In contrast, natural ponds at the 900 m scale had a smaller range of mean current values from -0.46 to -0.13 (mean = − 0.20 ± 0.041 SE) and the number of nearest neighbours ranged from 12 to 141 (mean = 94 ± 13.37, Fig. 5). At the 300 m scale, mean current density values at stormwater ponds ranged from − 1.15 to − 0.26 (mean = − 0.70 ± 0.036 SE) and the number of nearest neighbours ranged from one to six (mean = 2 ± 0.24 SE, Fig. 5). Natural ponds at the 300 m scale had a range of mean current values from − 0.40 to − 0.05 (mean = − 0.24 ± 0.041 SE) and the number of nearest neighbours ranged from 3 to 20 (mean = 11 ± 1.75 SE, Fig. 5). Overall, we found that natural ponds had significantly higher mean current and more nearest neighbours at both spatial scales measured (Fig. 5).

## Discussion

We hypothesized that connectivity surrounding both natural and stormwater ponds would influence odonate abundance, species richness, Shannon diversity, and species composition and that natural ponds would have higher levels of connectivity than stormwater ponds. Our results partially support our hypotheses. Dragonfly (Anisoptera) estimated adult abundance and species richness were both positively affected by increased levels of connectivity and number of nearest neighbours, however, there was no association found with Shannon diversity. We found that damselflies (Zygoptera) were largely unaffected by connectivity levels and number of nearest neighbours at the scale tested, with only one statistically significant result, a slight negative relationship between species richness and connectivity. Species composition of dragonflies and damselflies were significantly related to both connectivity and number of nearest neighbours. However, the species composition analysis revealed that there was only one species that was strongly associated with mean current density and number of nearest neighbours, *Leucorrhinia frigida,* the frosted whiteface, which was positively associated with both connectivity measures. The frosted whiteface was found almost exclusively at natural ponds and had very high abundances at natural ponds. As anticipated, we found that connectivity around stormwater ponds was reduced because of lower quality surrounding land cover types compared to natural ponds.

### Dragonflies

Dragonfly abundance had the strongest relationship observed with connectivity. Connectivity and number of nearest neighbours were both positively associated with dragonfly estimated abundance. Dragonflies are generally considered insects with strong flying capabilities and are active dispersers, having the ability to choose to disperse to the next pond if their visual cues indicate it may be suitable breeding habitat (Osborn and Samways 1996; Wikelski et al. 2006; McCauley 2007). Hostile land cover types, especially roads, pose serious threats to odonate survival during dispersal (Soluk et al. 2011). For example, 1261 odonate mortalities were recorded on a 2-km stretch of an Ontario highway over two summers (Baxter-Gilbert et al. 2015). Indeed, roads are widely regarded to act as hostile infrastructure and cause significant mortality in large mammals (Popp et al. 2014; Moore et al. 2023), birds (Erritzoe et al. 2003), and insects generally (Muñoz et al. 2015). When connectivity and number of nearest neighbours increase, this creates corridors of safe land cover types that allow for safer dispersal and increases the chance of animals including dragonflies encountering new habitats on their dispersal path (Pedruski and Arnott 2011; Hill et al. 2018). The creation of safe corridors through connectivity supports the positive relationship we found between connectivity and number of nearest neighbours with estimated abundance of dragonflies.

Dragonfly species richness was positively affected by connectivity, but less so than abundance. Both mean current density and number of nearest neighbours had a positive relationship with dragonfly species richness. The increased ease of dispersal coupled with the higher likelihood of encountering other suitable habitats increases the number of species present at each pond (Brown et al. 2016). The potential for metapopulation within an urban environment, a traditionally fragmented and risky landscape, increases the chances of regional persistence of dragonfly species and promotes ecosystem health (Razeng et al. 2016; Hill et al. 2017). The presence of multiple pond types, including man-made ponds, can promote high biodiversity of odonates by creating ponds at different levels of succession, providing more environmental conditions and increasing connectivity (Dolný and Harabiš 2012). Our current map shows that there are connectivity pathways, facilitated by urban ponds acting as stepping stones for dragonflies when present in high enough numbers. This stepping stone effect influencing species composition can be seen by the strong association between connectivity and *Leucorrhinia frigida,* the frosted whiteface, which was positively associated with both connectivity measures. The frosted whiteface occurred at natural ponds with high levels of mean current and number of nearest neighbours. A study of mine subsidence in Czech Republic found that a dragonfly from the same genus, *Leucorrhinia pectoralis*, formed metapopulations as an adaptation to frequent freshwater disturbance (Dolný and Harabiš 2012). Further, other *Leucorrhinia* species have shown to take flight less often than other species when presented with undesirable land cover types, i.e., dense forest (French and McCauley 2019) and prefer open land cover types when traversing distances under 700 m (Chin and Taylor 2009). The genus’ preference for easily traversable land cover types and avoidance of dispersal when preferred land over types are not present, may explain why *Leucorrhinia frigida* was the dragonfly species with the strongest association with mean current density and number of nearest neighbours. The presence of *Leucorrhinia frigida* in almost exclusively natural ponds that have very high levels of connectivity, supports the power of the stepping stone effect. However, if not all stormwater ponds are well connected, it may be difficult for species found in areas with natural wetland complexes to spread throughout the city. Urban ponds benefit from dragonfly richness and abundance, as dragonflies occupy a key trophic level acting as both prey and predator species for a variety of taxa (Corbet 1999). Therefore, larger odonate populations as well as higher levels of odonate species richness can indicate the ability of the ecosystem to support other trophic levels (Bried et al. 2007).

### Damselflies

Damselflies did not appear to have strong relationships with connectivity at the scale we measured. There were no significant relationships between damselfly abundance and connectivity. Although there was a significant relationship between species richness and mean current density, the relationship was negative and explained only 11% of the variation. Damselflies are typically weak fliers which stay close to their natal habitat (McPeek 1989), which could explain the lack of relationships. In addition, a high amount of variation in damselfly community composition has been attributed to more site-specific factors such as plant community structure due to their nature as obligate endophytes (i.e., they lay their eggs in plant tissue, Perron and Pick 2020a). The negative relationship between damselfly species richness and mean current density may be driven by the high richness and abundance of dragonflies at the ponds with high connectivity. Dragonflies are known predators of damselflies; a field experiment reported up to 80% mortality of damselfly nymphs in the presence of two dragonfly nymph species (Wissinger and McGrady 1993). Another observational study concluded that dragonfly nymphs were the most abundant invertebrate predator for the damselfly genus *Enallagma* in Michigan lakes and that dragonfly nymphs affected the distributional patterns of the damselflies (McPeek 1990). Thus, the inverse relationship between dragonflies and damselflies with connectivity and the relationships with species composition may be a result of predator–prey relations, with increased predation pressures on damselflies in ponds with high abundances of dragonflies likely facilitated by increased connectivity for dragonflies.

### Shannon diversity

We did not find strong relationships between Shannon diversity and connectivity for dragonflies and damselflies. Diversity and species composition of odonates has been previously explained by environmental variables such as plant species composition, water quality, and habitat size (Le Gall et al. 2018), which collectively can explain between 37 and 52% of variation in odonate species composition (Perron et al. 2021). The biodiversity of urban pond aquatic invertebrates, reptiles, amphibians, and fishes can be tied to both connectivity and pond-level characteristics such as pond intermittency and presence of predators (Hyseni et al. 2021; Trovillion et al. 2022). A habitat with a range of different local environmental conditions, such as plant species composition and substrates, which odonates use for specific life history requirements and behaviours, allows for the occupation of niches (de Resende et al. 2021). In addition, the lack of an association between Shannon diversity and connectivity in natural wetlands may be a result of the sampling landscape. Due to the nature of modern urban development, many naturally occurring wetlands are dried and/or built over in the process of urbanization (Alikhani et al 2021). Consequently, natural wetlands can be difficult to find in cities. Our selection of natural ponds included ponds that were a similar size to stormwater ponds (~ 1 ha) and had < 1% of impervious cover within their catchment area. These requirements were limiting and resulted in sampling a relatively low number of natural ponds, and several natural ponds that were close together in the landscape. Although the clustering of natural ponds is a result of natural processes and is representative of the landscape, it is also a limitation of our study. The clustering of our natural ponds means that we may not be assessing odonate trends on a large scale and are instead just getting a picture of one specific area on the landscape.

### Natural vs stormwater ponds

We found that natural ponds consistently had significantly higher levels of connectivity than stormwater ponds. This finding was expected, as the very nature of stormwater ponds means they are integrated into urbanized areas, largely residential areas in our study, if they are to fulfill their purpose of mitigating runoff from impervious surfaces. By contrast, the natural ponds in our study were in more rural, less developed landscapes that have retained the natural pond network and surrounding forest. The disparity we observed between natural and stormwater ponds shows that natural wetlands cannot be replaced by constructed wetlands, with connectivity being one of many reasons for this. However, we did find that a subset of the urban stormwater ponds were close to having the same level of mean current and number of nearest neighbours as some of the natural ponds, indicating that it is possible to build highly connected habitats within the urban landscape. Further, there were four stormwater ponds that rivalled the abundance of natural ponds with dragonfly estimated abundance counts around 100, even though the number of nearest neighbours for stormwater ponds was much lower than natural ponds. The four stormwater ponds with the most dragonfly abundance all had nine or more potential habitats within 900 m and mean current density values on or above the median (− 0.67). There were stormwater ponds that had similar dragonfly species richness values when compared to natural ponds, which is consistent with the literature. There are many factors that influence dragonfly species richness at stormwater ponds, including plant community, pond design, and pond water quality (Johansson et al. 2019; Perron and Pick 2020a, b).

## Conclusion

We demonstrate that improved connectivity has the potential to increase abundance and species richness of adult dragonflies in urban areas. Estimates of dragonfly abundance and species richness increased with higher connectivity and number of nearest neighbours. Damselflies were largely unaffected by connectivity and habitat levels which supports previous literature suggesting that damselflies have high fidelity to their natal ponds. We found that stormwater ponds consistently had lower connectivity and number of nearest neighbours when compared to their natural pond counterparts. The standard approach of designing and managing our urban blue and green spaces is likely not enough to support Odonata metapopulation dynamics. We recommend intentional planning of urban stormwater pond networks, with individual ponds acting as stepping-stones within dispersal distance of odonates, to promote larger population sizes and the creation of metacommunities within the city. Further, low resistance land cover types, such as grasslands and other green spaces, could be placed strategically in the surrounding landscape to facilitate connectivity and improve species richness and estimated abundance of dragonflies, and potentially other dispersing wetland species, in urban areas.

### Supplementary Information

Below is the link to the electronic supplementary material.

Supplementary file1 (PDF 43 KB)

Supplementary file2 (PDF 82 KB)

Supplementary file3 (PDF 108 KB)

Supplementary file4 (PDF 164 KB)

## Data Availability

The data and code generated during and/or analysed during the current study is archived and published on Zenodo at https://zenodo.org/records/5347801 and GitHub and can be found at github.com/icrichmond/OdonataConnectivity.
