# Supplementary material for: Connectivity of stormwater ponds impacts Odonata abundance and species richness
Source: Landsc Ecol. 2024 Feb 28;39(3):63. doi: 10.1007/s10980-024-01817-z (PMC10902110; doi:10.1007/s10980-024-01817-z)
Supplement: Supplementary file 1 — Supplementary file1 (PDF 43 KB) [file 10980_2024_1817_MOESM1_ESM.pdf]

Title: Connectivity of stormwater ponds impacts Odonata abundance and species richness

Journal: Landscape Ecology

Authors: Richmond, Isabella C.*, Perron, Mary Ann C., Boyle, Sean B., & Pick, Frances R.

* Department of Biology, 30 Marie Curie Private, University of Ottawa, Ottawa, Ontario K1N 6N5, Canada Department of Biology, 7141 Sherbrooke St. W., Concordia University, Montreal, QC, Canada, H4B 1R6

email: isabella.richmond@mail.concordia.ca, phone : 438-439-8064

Supplementary Information

This Supplementary Information provides fine-scale details for each of our study ponds. Table S1-1 outlines descriptive characteristics of each pond, like age and area. Table S1-2 details the observed odonate data at each pond, including abundance, species richness, and shannon diversity. Table S1-3 describes the connectivity data at each pond, including mean and standard variation for current values, as well as number of nearest neighbours. All code and data for this project can be found at https://github.com/icrichmond/OdonataConnectivity.

Table S1-1. Pond descriptive characteristics. SWF = stormwater pond, NAT = natural pond. Coordinates are in WGS 84. Some data were not available for each pond, indicated by NA.

Pond	Group	X coordinate	Y coordinate	Age (years)	Area (m2)	Catchment Area (km2)	Imperviousness of Catchment (%)
SWF-1127	SWF	45.33017974,	-75.72884	32	10063	1.471	59.9900
SWF-1133	SWF	45.36830783,	-75.73527	16	2725	0.373	55.0500
SWF-1134	SWF	45.31763596,	-75.78754	21	860	0.066	48.5800
SWF-1204	SWF	45.34220373,	-75.90695	19	2935	0.100	74.3400
SWF-1205	SWF	45.32004202,	-75.88933	19	3306	0.452	40.1600
SWF-1206	SWF	45.31482398,	-75.89826	16	8800	0.460	41.7500
SWF-1207	SWF	45.31016948,	-75.92454	17	11191	0.745	44.2000
SWF-1208	SWF	45.30832635,	-75.92351	21	6799	0.562	67.7700
SWF-1209	SWF	45.31152481,	-75.93422	13	2578	0.215	39.5100
SWF-1215	SWF	45.35286907,	-75.92465	11	3057	1.353	46.4100
SWF-1220	SWF	45.41657243,	-76.14761	9	578	0.032	NA
SWF-1221	SWF	45.3475836,	-76.03235	12	1081	0.078	42.1700
SWF-1227	SWF	45.35740207,	-75.93654	14	8154	0.441	45.8900
SWF-1228	SWF	45.34245843,	-75.93618	9	7974	0.197	50.4100
SWF-1234	SWF	45.36322865,	-75.92050	8	5244	0.087	39.4900
SWF-1235	SWF	45.36465947,	-75.92308	8	5236	0.259	51.3900
SWF-1236	SWF	45.35621585,	-75.93270	6	1914	0.182	64.7200
SWF-1253	SWF	45.29948854,	-75.93429	3	2131	0.210	21.9900
SWF-1306	SWF	45.2854921,	-75.88746	13	5267	0.695	36.2800
SWF-1310	SWF	45.27005163,	-75.94333	15	3334	0.447	41.1000
TIM-2	SWF	45.26972619,	-75.94557	15	5867	NA	NA
SWF-1311	SWF	45.27185572,	-75.94189	14	3512	0.087	47.6900
SWF-1312	SWF	45.25775185,	-75.93189	21	2761	0.909	44.8000
SWF-1314	SWF	45.26769323,	-75.91224	14	7804	0.419	44.0900
SWF-1316	SWF	45.29861425,	-75.91925	23	4474	0.483	73.2900
SWF-1320	SWF	45.2738277,	-75.91380	18	1143	0.233	45.1800
SWF-1325	SWF	45.27357088,	-75.90202	5	10311	0.454	44.5700
SWF-1347	SWF	45.2860605,	-75.90843	6	1553	0.040	62.4600
SWF-1352	SWF	45.27847676,	-75.87882	2	13823	NA	NA
SWF-1428	SWF	45.27107658,	-75.77662	4	10415	NA	NA
SWF-1439	SWF	45.29284369,	-75.79058	23	5805	0.140	8.2600
SWF-1444	SWF	45.26420368,	-75.78128	1	14798	NA	NA
SWF-1501	SWF	45.13916237,	-75.71950	36	1267	0.157	25.1900
SWF-1610	SWF	45.3512119,	-75.64519	22	2905	0.157	46.1400
SWF-1616	SWF	45.36931473,	-75.65284	13	2996	0.502	33.6900
SWF-1621	SWF	45.34360175,	-75.62729	12	3506	0.315	48.1400

SWF-1622	SWF	45.40248832,	-75.63949	22	2765	0.210	40.3600
SWF-1802	SWF	45.26743492,	-75.59548	10	2450	0.186	41.7300
SWF-1808	SWF	45.23123898,	-75.59896	11	1060	0.018	11.5300
SWF-1809	SWF	45.23180344,	-75.59808	11	722	0.054	11.5400
SWF-1914	SWF	45.46813473,	-75.44681	12	8268	0.033	NA
NAT-1	NAT	45.33810824,	-75.94408	NA	1908	0.483	0.0000
NAT-2	NAT	45.38493452,	-76.07759	NA	4750	2.142	0.0050
NAT-3	NAT	45.04808296,	-75.85890	NA	4874	1.001	0.0080
NAT-6	NAT	45.38193056,	-76.08365	NA	2963	2.954	0.0020
NAT-7	NAT	45.39146021,	-76.07134	NA	7999	2.142	0.0050
NAT-9	NAT	45.38772056,	-76.07981	NA	1586	2.081	0.0003
NAT-10	NAT	45.38854686,	-76.08141	NA	9379	2.081	0.0003
NAT-11	NAT	45.388351	-76.07052	NA	3641	NA	NA

Table S1-2. Pond odonate data. All sampling was done on adult odonates. SWF = stormwater pond, NAT = natural pond.

Pond	Group	Dragonfly Abundance	Dragonfly Species Richness	Dragonfly Shannon Diversity	Damselfly Abundance	Damselfly Species Richness	Damselfly Shannon Diversity
SWF-1127	SWF	29	8	1.8459874	259	7	1.0925162
SWF-1133	SWF	15	5	1.3378607	91	8	1.5391545
SWF-1134	SWF	7	1	0.0000000	2	2	0.6931472
SWF-1204	SWF	31	6	1.1310939	323	5	1.2076280
SWF-1205	SWF	51	7	1.5041688	115	8	1.5328597
SWF-1206	SWF	59	8	1.6309058	177	9	1.5242646
SWF-1207	SWF	12	4	1.1269288	156	5	0.4424649
SWF-1208	SWF	52	9	1.7334709	203	4	1.1251037
SWF-1209	SWF	104	8	1.1786722	534	6	1.2228198
SWF-1215	SWF	53	8	1.2800596	144	4	1.0692064
SWF-1220	SWF	37	10	1.8955167	54	4	1.1637602
SWF-1221	SWF	106	5	0.8329993	307	6	1.0617073
SWF-1227	SWF	8	4	1.0735428	149	9	1.2665411
SWF-1228	SWF	102	12	1.9134992	99	6	1.1760296
SWF-1234	SWF	69	9	1.5226852	707	8	1.1283662
SWF-1235	SWF	60	8	1.6420220	274	7	1.1610703
SWF-1236	SWF	29	8	1.4313443	349	9	1.3403244
SWF-1253	SWF	17	5	1.2033320	119	8	0.7494566
SWF-1306	SWF	21	9	1.8179404	267	7	0.8355309
SWF-1310	SWF	33	9	1.8282090	388	8	1.5191383
TIM-2	SWF	39	8	1.6066887	379	11	1.6376775
SWF-1311	SWF	31	6	1.2916361	105	8	1.1647696
SWF-1312	SWF	37	7	1.5476067	153	10	1.5230521
SWF-1314	SWF	26	7	1.6242896	197	7	0.9210818
SWF-1316	SWF	29	9	1.9566326	102	7	1.1564823
SWF-1320	SWF	99	10	1.5453142	152	5	1.1796693
SWF-1325	SWF	6	3	0.8675632	82	6	0.8530181
SWF-1347	SWF	89	7	1.2219150	170	8	1.4307411
SWF-1352	SWF	70	11	2.1267632	581	7	0.7970905
SWF-1428	SWF	19	8	1.7494938	216	6	0.6000191
SWF-1439	SWF	19	9	1.9587271	31	3	1.0182776
SWF-1444	SWF	60	10	2.0865832	110	10	1.5356398
SWF-1501	SWF	73	7	1.5495872	137	6	1.3254806
SWF-1610	SWF	14	7	1.6681740	26	6	1.5612525
SWF-1616	SWF	7	4	1.1537419	82	7	0.9431038
SWF-1621	SWF	29	8	1.7500308	33	8	1.8887141
SWF-1622	SWF	4	2	0.5623351	55	8	1.2840984
SWF-1802	SWF	26	7	1.4555009	431	5	1.1415286
SWF-1808	SWF	44	10	1.7556630	88	9	1.5417784
SWF-1809	SWF	52	9	1.6796285	189	7	1.3016903
SWF-1914	SWF	24	11	2.0805708	100	5	0.7145236
NAT-1	NAT	100	4	0.6275549	15	3	0.4850941
NAT-2	NAT	290	11	0.5717056	612	5	0.3762368
NAT-3	NAT	135	10	1.4252790	337	6	1.6030965
NAT-6	NAT	236	10	1.2038193	99	7	1.3628141
NAT-7	NAT	304	11	1.1254583	96	5	1.1583630

NAT-9	NAT	165	11	1.6324814	73	4	0.7271346
NAT-10	NAT	251	11	1.2450483	130	5	0.9509359
NAT-11	NAT	169	9	0.7926793	155	5	0.8217469

Table S1-3. Pond connectivity data. SWF = stormwater pond, NAT = natural pond. 900 m scale was used for dragonflies, 300 m scale was used for damselflies. Higher values of mean current indicate higher connectivity. Current was calculated using *Circuitscape*.

Pond	Group	Mean Current Density (900 m)	Number of Nearest Neighbours (900 m)	Mean Current Density (300 m)	Number of Nearest Neighbours (300 m)
SWF-1127	SWF	-0.8541740	4	-0.80552565	2
SWF-1133	SWF	-0.9417346	1	-0.97233266	1
SWF-1134	SWF	-0.5252690	1	-0.45472682	1
SWF-1204	SWF	-0.6722568	7	-0.66845248	1
SWF-1205	SWF	-0.7059275	2	-0.72230617	1
SWF-1206	SWF	-0.8476087	4	-0.66576810	2
SWF-1207	SWF	-0.6868062	9	-0.69059380	2
SWF-1208	SWF	-0.6528352	10	-0.37240946	4
SWF-1209	SWF	-0.5330535	9	-0.29624758	3
SWF-1215	SWF	-0.8604614	7	-0.84148678	3
SWF-1220	SWF	-0.3443741	2	-0.43221126	1
SWF-1221	SWF	-0.6658585	12	-0.93108203	3
SWF-1227	SWF	-0.7548048	6	-0.81682141	2
SWF-1228	SWF	-0.7165849	27	-0.72614397	5
SWF-1234	SWF	-0.6646492	8	-0.55812583	4
SWF-1235	SWF	-0.6222274	8	-0.52577686	4
SWF-1236	SWF	-0.8364039	7	-0.81066658	2
SWF-1253	SWF	-0.7030652	2	-0.69122542	1
SWF-1306	SWF	-0.6018423	8	-0.44917477	4
SWF-1310	SWF	-0.6752789	29	-0.90676935	5
TIM-2	SWF	-0.6174634	26	-0.94674093	4
SWF-1311	SWF	-0.6914392	29	-0.74430770	6
SWF-1312	SWF	-0.8303875	27	-0.96843916	1
SWF-1314	SWF	-0.8215422	13	-0.82253174	3
SWF-1316	SWF	-0.5441649	9	-0.26295161	3
SWF-1320	SWF	-0.8548104	11	-0.79052566	2
SWF-1325	SWF	-0.6289223	4	-0.82785151	2
SWF-1347	SWF	-0.4897167	12	-0.47834002	3
SWF-1352	SWF	-0.5248569	3	-0.42611619	1
SWF-1428	SWF	-0.8405755	1	-1.05200309	1
SWF-1439	SWF	-0.5473483	8	-0.46688144	1
SWF-1444	SWF	-0.5343336	4	-0.36732638	2
SWF-1501	SWF	-0.5595385	1	-0.72975220	1
SWF-1610	SWF	-1.0544679	11	-1.14614714	5
SWF-1616	SWF	-0.9669136	3	-0.86036813	1
SWF-1621	SWF	-0.8113038	20	-0.91059999	2
SWF-1622	SWF	-0.9661279	2	-1.06753968	1
SWF-1802	SWF	-0.5198229	45	-0.67053335	6
SWF-1808	SWF	-0.4568623	20	-0.40997693	4
SWF-1809	SWF	-0.4636718	18	-0.41361987	4
SWF-1914	SWF	-0.6988018	25	-0.94413676	1
NAT-1	NAT	-0.4613936	59	-0.39600918	12
NAT-2	NAT	-0.1276601	107	-0.04651158	8
NAT-3	NAT	-0.2661014	23	-0.30351249	3
NAT-6	NAT	-0.1741004	102	-0.17032987	20

NAT-7	NAT	-0.1526200	96	-0.30863477	10
NAT-9	NAT	-0.1338795	130	-0.13961485	11
NAT-10	NAT	-0.1289999	141	-0.19905078	14
NAT-11	NAT	-0.1319091	96	-0.32746350	14
