# Supplementary material for: Connectivity of stormwater ponds impacts Odonata abundance and species richness
Source: Landsc Ecol. 2024 Feb 28;39(3):63. doi: 10.1007/s10980-024-01817-z (PMC10902110; doi:10.1007/s10980-024-01817-z)
Supplement: Supplementary file 2 — Supplementary file2 (PDF 82 KB) [file 10980_2024_1817_MOESM2_ESM.pdf]

Title: Connectivity of stormwater ponds impacts Odonata abundance and species richness

Journal: Landscape Ecology

Authors: Richmond, Isabella C.*, Perron, Mary Ann C., Boyle, Sean B., & Pick, Frances R.

* Department of Biology, 30 Marie Curie Private, University of Ottawa, Ottawa, Ontario K1N 6N5, Canada
Department of Biology, 7141 Sherbrooke St. W., Concordia University, Montreal, QC, Canada, H4B 1R6
email: isabella.richmond@mail.concordia.ca, phone : 438-439-8064

Supplementary Information

This supplementary file contains the model summary output tables for the statistically significant models presented in the paper. We used general linear models to test the relationships between our variables. After running general linear models with all three explanatory variables, we determined that standard deviation of current was acting as an uninformative parameter and was thus removed from all analysis. Mean current and number of surrounding habitats were highly correlated, so two separate models were run for every response variable. Estimated abundance, shannon diversity, and species richness were each modelled with mean current and number of surrounding habitats separately at the 900 m and 300 m scales, resulting in a total of 12 models (six for dragonflies and six for damselflies). The "lm" function with a Gaussian error structure was used to execute our models. Residuals were checked for assumptions of normality, independence, and homogeneity before analyzing our results. To see visualization of the relationships please refer to the main paper.

Table 1. Effect of mean current and number of surrounding habitats on dragonfly estimated abundance and species richness based on general linear models. Mean current and number of surrounding habitats were calculated at a 900 m scale surrounding the study sites. Two separate models were used because mean current and number of surrounding habitats are highly correlated. Models use a normal error distribution.

	Estimate	Standard Error	t-value	p-value
**Estimated Abundance**				
Intercept	209.40	18.75	11.17	$8.02x10^{-15}$
Mean Current	231.66	28.66	8.08	$1.92x10^{-10}$
Intercept	25.81	7.04	3.67	$6.21x10^{-4}$
Number of Surrounding Habitats	1.72	0.16	10.48	$6.94x10^{-14}$
**Species Richness**				
Intercept	10.78	0.90	11.95	$7.52x10^{-16}$
Mean Current	4.97	1.38	3.60	$7.59x10^{-04}$
Intercept	7.00	0.41	17.09	$8.41x10^{-22}$
Number of Surrounding Habitats	0.031	$9.54x10^{-03}$	3.20	$2.47x10^{-3}$

Table 2. Summary of damselfly general linear models. Damselfly Shannon diversity and species richness are the dependent variables, with mean current and number of surrounding habitats as the independent variables. Mean current was calculated at a 300 m scale surrounding the study sites. Models use a normal error distribution.

	Estimate	Standard Error	t-value	p-value
**Shannon Diversity**				
Intercept	0.94	0.12	7.95	3.00×10^{-10}
Mean Current	-0.32	0.17	-1.84	0.071
**Species Richness**				
Intercept	5.02	0.66	7.59	1.05×10^{-09}
Number of Surrounding Habitats	-2.40	0.97	-2.47	0.017
