# Supplementary material for: Connectivity of stormwater ponds impacts Odonata abundance and species richness
Source: Landsc Ecol. 2024 Feb 28;39(3):63. doi: 10.1007/s10980-024-01817-z (PMC10902110; doi:10.1007/s10980-024-01817-z)
Supplement: Supplementary file 3 — Supplementary file3 (PDF 108 KB) [file 10980_2024_1817_MOESM3_ESM.pdf]

Title: Connectivity of stormwater ponds impacts Odonata abundance and species richness

Journal: Landscape Ecology

Authors: Richmond, Isabella C.*, Perron, Mary Ann C., Boyle, Sean B., & Pick, Frances R.

* Department of Biology, 30 Marie Curie Private, University of Ottawa, Ottawa, Ontario K1N 6N5, Canada
Department of Biology, 7141 Sherbrooke St. W., Concordia University, Montreal, QC, Canada, H4B 1R6
email: isabella.richmond@mail.concordia.ca, phone : 438-439-8064

Supplementary Information

This supplementary file contains the transformation-based redundancy analyses of the damselfly species composition data with the mean current and the number of surrounding habitats. Adult damselfly species composition was tested for relationships with mean current at a 300 m scale. The mean current at a 300 m scale was significantly associated with damselfly species composition (R^2 = 0.06, p = 0.002). The RDA model (p = 0.004) and axis (p= 0.001) were significant. The proportion of unconstrained variation again was substantially higher than the proportion of constrained variation that can be explained by the model, 0.31 and 0.08 respectively. Adult damselfly species composition was also significantly associated with the number of surrounding habitats at a 300 m scale (R^2 = 0.11, p = 0.002). The RDA model (p = 0.001) and axis (p = 0.001) were significant. The proportion of unconstrained variation again was substantially higher than the proportion of constrained variation explained by the model, 0.30 and 0.04 respectively.

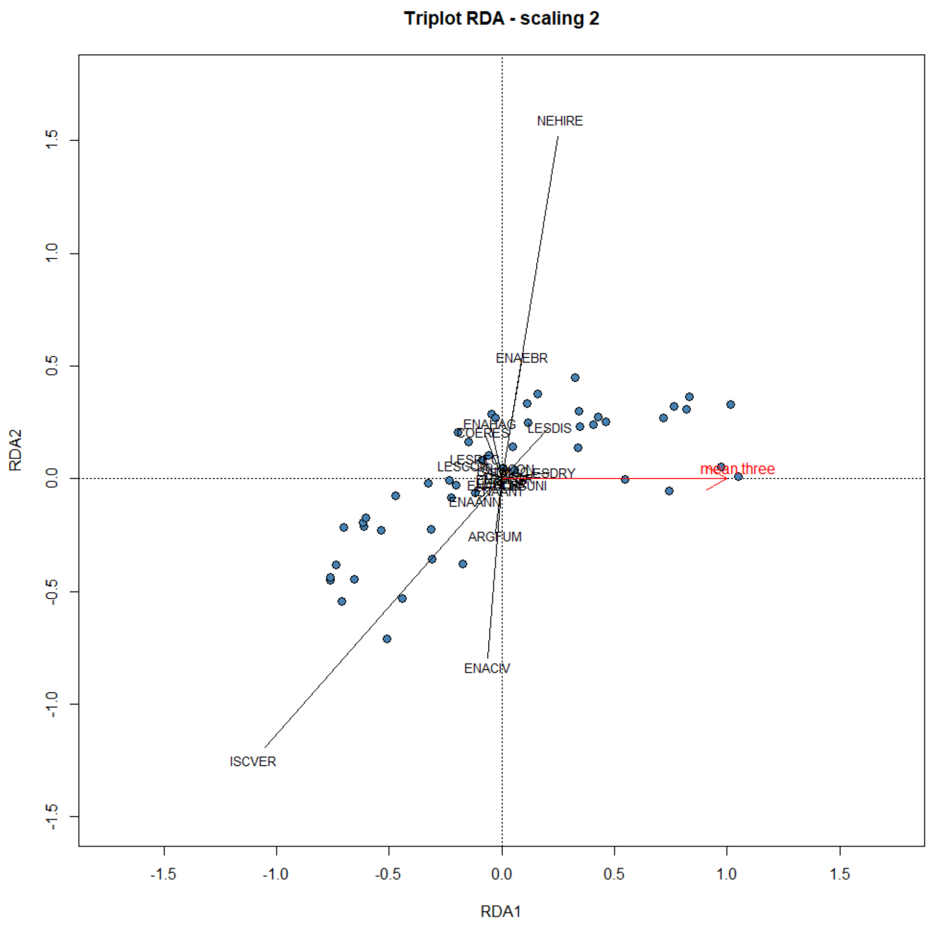

Figure S4-1. Transformation based-redundancy analysis (tb-rda), using a Hellinger transformation, of the relationship between adult damselfly species composition (black vectors without arrows) and mean current at a 300m scale (mean.three) at urban ponds (n = 49).

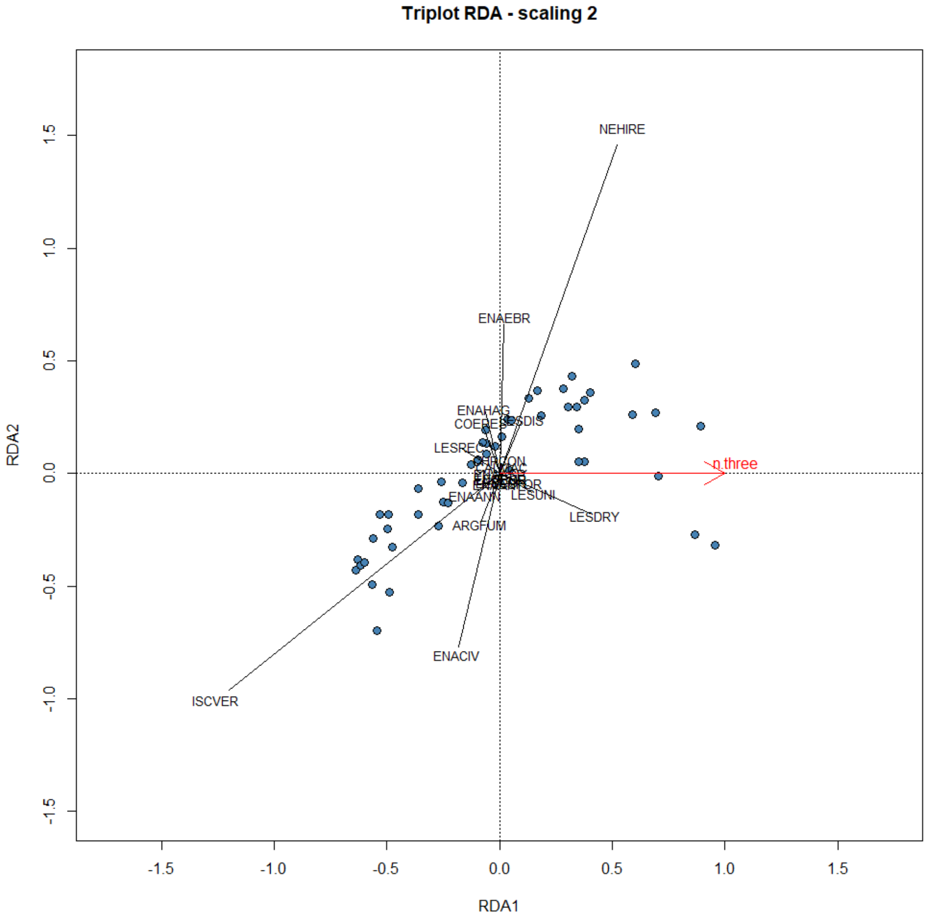

Figure S4-2. Transformation based-redundancy analysis (tb-rda), using a Hellinger transformation, of the relationship between adult damselfly species composition (black vectors without arrows) and the number of surrounding habitats at a 300m scale (n.three) at urban ponds (n = 49).
