# Supplementary material for: Connectivity of stormwater ponds impacts Odonata abundance and species richness
Source: Landsc Ecol. 2024 Feb 28;39(3):63. doi: 10.1007/s10980-024-01817-z (PMC10902110; doi:10.1007/s10980-024-01817-z)
Supplement: Supplementary file 4 — Supplementary file4 (PDF 164 KB) [file 10980_2024_1817_MOESM4_ESM.pdf]

Title: Connectivity of stormwater ponds impacts Odonata abundance and species richness

Journal: Landscape Ecology

Authors: Richmond, Isabella C.*, Perron, Mary Ann C., Boyle, Sean B., & Pick, Frances R.

* Department of Biology, 30 Marie Curie Private, University of Ottawa, Ottawa, Ontario K1N 6N5, Canada
Department of Biology, 7141 Sherbrooke St. W., Concordia University, Montreal, QC, Canada, H4B 1R6
email: isabella.richmond@mail.concordia.ca, phone : 438-439-8064

Supplementary Information

This supplementary file contains the species name acronyms as well as transformation-based redundancy analyses of the damselfly species composition data with the mean current and the number of surrounding habitats. Adult damselfly species composition was tested for relationships with mean current at a 300 m scale. The mean current at a 300 m scale was significantly associated with damselfly species composition ($R^2 = 0.06$, $p = 0.002$). The RDA model ($p = 0.004$) and axis ($p= 0.001$) were significant. The proportion of unconstrained variation again was substantially higher than the proportion of constrained variation that can be explained by the model, 0.31 and 0.08 respectively. Adult damselfly species composition was also significantly associated with the number of surrounding habitats at a 300 m scale ($R^2 = 0.11$, $p = 0.002$). The RDA model ($p = 0.001$) and axis ($p = 0.001$) were significant. The proportion of unconstrained variation again was substantially higher than the proportion of constrained variation explained by the model, 0.30 and 0.04 respectively.

Table S2. Odonata (Zygoptera and Anisoptera) species at urban stormwater ponds (SWP, n=41) and natural ponds (NAT, n=8) with abbreviations used in Fig. 3, Fig S4-1, and Fig S4-2.

Scientific name	Abbreviation
Suborder: Zygoptera	
Calopteryx maculata	CALMAC
Lestes congener	LESCON
Lestes disjunctus	LESDIS
Lestes dryas	LESDRY
Lestes eurinus	LESEUR
Lestes rectangularis	LESREC
Lestes unguiculatus	LESUNI
Argia fumipennis violacea	ARGFUM
Chromagrion conditum	CHRCON
Coenagrion resolutum	COERES
Enallagma annexum	ENAANN
Enallagma antennatum	ENAANT
Enallagma aspersum	ENAASP
Enallagma boreale	ENABOR
Enallagma civile	ENACIV
Enallagma ebrium	ENAEBR
Enallagma hageni	ENAHAG
Ischnura verticalis	ISCVER
Nehalennia irene	NEHIRE
Suborder: Anisoptera	
Aeshna canadensis	AESCAN
Aeshna constricta	AESCON
Aeshna tuberculifera	AESTUB
Aeshna umbrosa	AESUMB
Anax junius	ANAJUN
Arigomphus cornutus	ARICOR
Dromogomphus spinosus	DROSPI
Epitheca cynosura	EPICYN
Epitheca canis	EPICAN
Epitheca princeps	EPIPRI
Cordulia shurtleffi	CORSHU
Dorocordulia libera	DORLIB
Celithemis elisa	CELELI
Erythemis simplicicollis	ERYSIM
Leucorrhinia frigida	LEUFRI
Leucorrhinia intacta	LEUINT
Leucorrhinia proxima	LEUPRO
Pachydiplax longipennis	PACLON
Perithemis tenera	PERTEN
Ladona julia	LADJUL
Libellula luctuosa	LIBLUC
Libellula pulchella	LIBPUL
Libellula quadrimaculata	LIBQUA
Plathemis lydia	PLALYD
Sympetrum costiferum	SYMCOS
Sympetrum internum	SYMINT
Sympetrum obtrusum	SYMOBT
Sympetrum semicinctum	SYMSEM
Sympetrum vicinum	SYMVIC

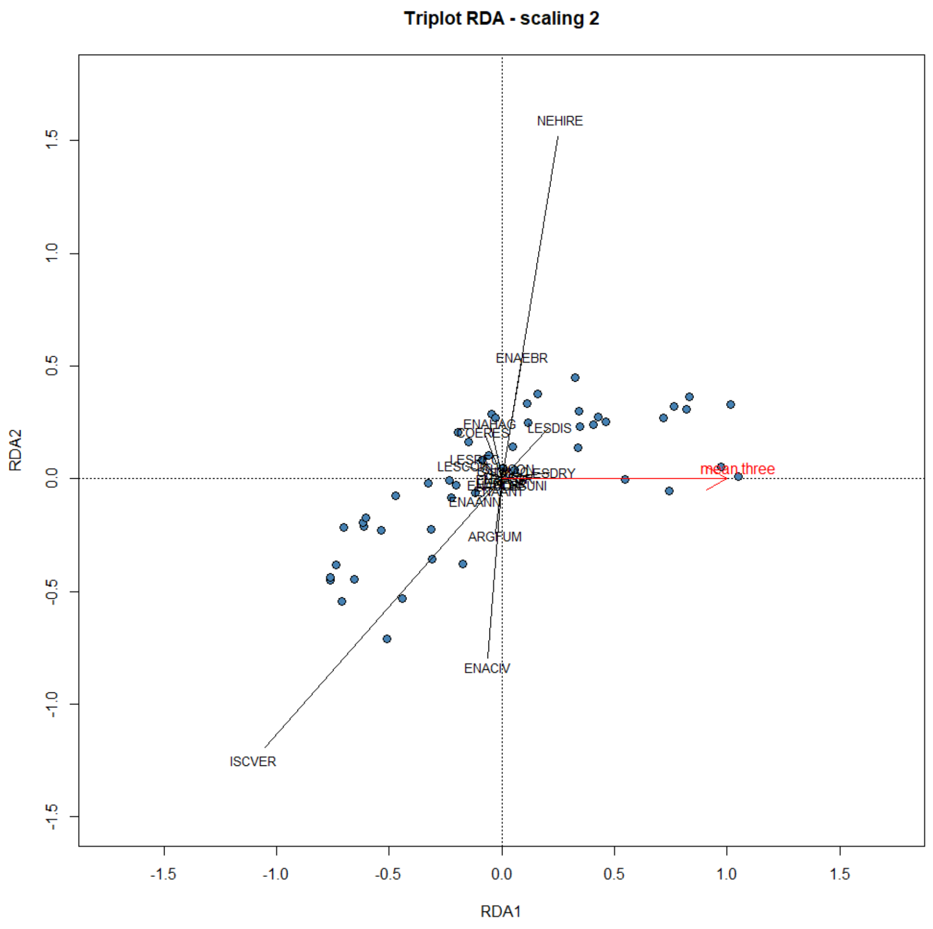

Figure S4-1. Transformation based-redundancy analysis (tb-rda), using a Hellinger transformation, of the relationship between adult damselfly species composition (black vectors without arrows) and mean current at a 300m scale (mean.three) at urban ponds (n = 49).

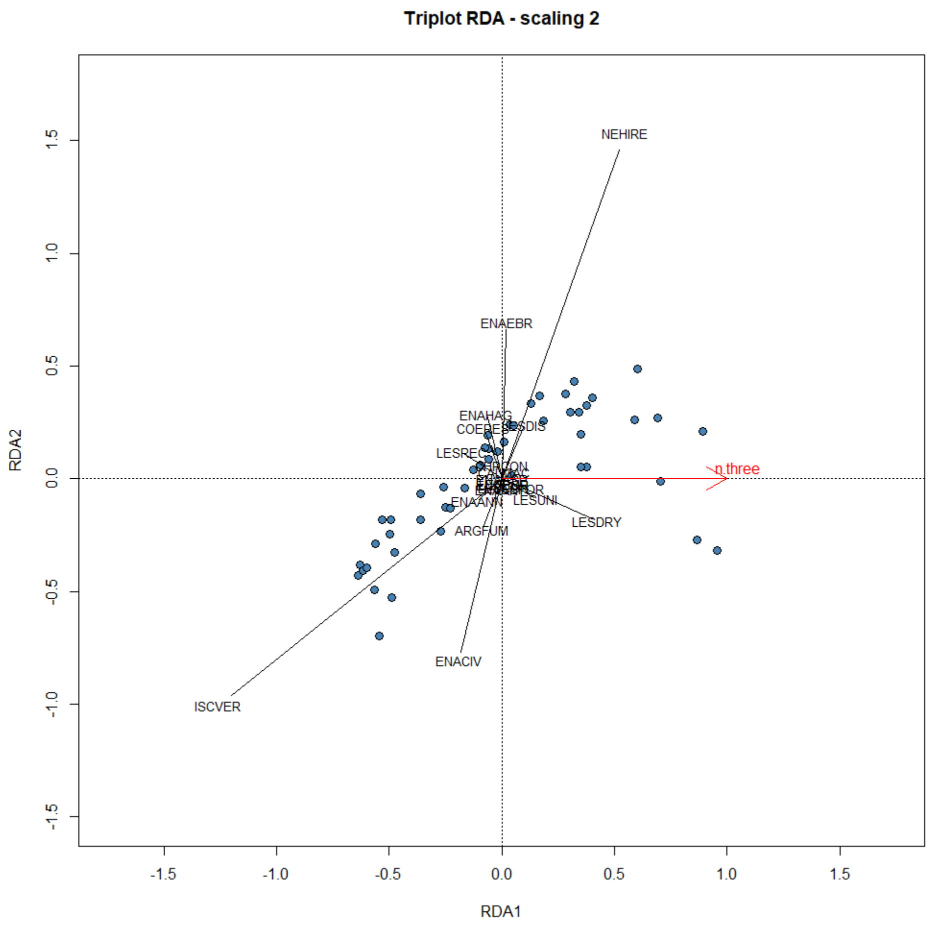

Figure S4-2. Transformation based-redundancy analysis (tb-rda), using a Hellinger transformation, of the relationship between adult damselfly species composition (black vectors without arrows) and the number of surrounding habitats at a 300m scale (n.three) at urban ponds (n = 49).
